# Evaluation of a Rapid Diagnostic Test for Detection of Burkholderia pseudomallei in the Lao People's Democratic Republic

**DOI:** 10.1128/JCM.02002-17

**Published:** 2018-06-25

**Authors:** Kate L. Woods, Latsaniphone Boutthasavong, Caoimhe NicFhogartaigh, Sue J. Lee, Viengmon Davong, David P. AuCoin, David A. B. Dance

**Affiliations:** a Lao-Oxford-Mahosot Hospital-Wellcome Trust Research Unit, Microbiology Laboratory, Mahosot Hospital, Vientiane, Lao PDR; b Bart's Health Division of Infection, Pathology and Pharmacy Department, Royal London Hospital, London, United Kingdom; c Centre for Tropical Medicine and Global Health, University of Oxford, Oxford, United Kingdom; d Mahidol-Oxford Tropical Medicine Research Unit, Mahidol University, Bangkok, Thailand; e Department of Microbiology and Immunology, University of Nevada, Reno School of Medicine, Reno, Nevada, USA; f Faculty of Infectious and Tropical Diseases, London School of Hygiene and Tropical Medicine, London, United Kingdom

**Keywords:** B. pseudomallei, melioidosis, blood, lateral flow immunoassay, pus, rapid diagnosis, serum, sputum, sterile fluid, urine

## Abstract

Burkholderia pseudomallei causes significant global morbidity and mortality, with the highest disease burden in parts of Asia where culture-based diagnosis is often not available. We prospectively evaluated the Active Melioidosis Detect (AMD; InBios International, USA) lateral flow immunoassay (LFI) for rapid detection of B. pseudomallei in turbid blood cultures, pus, sputum, sterile fluid, urine, and sera. The performance of this test was compared to that of B. pseudomallei detection using monoclonal antibody latex agglutination (LA) and immunofluorescence assays (IFA), with culture as the gold standard. AMD was 99% (99/100; 95% confidence interval, 94.6 to 100%) sensitive and 100% (308/308; 98.8 to 100%) specific on turbid blood culture bottles, with no difference from LA or IFA. AMD specificity was 100% on pus (122/122; 97.0 to 100%), sputum (20/20; 83.2 to 100%), and sterile fluid (44/44; 92 to 100%). Sensitivity on these samples was as follows: pus, 47.1% (8/17; 23.0 to 72.2%); sputum, 33.3% (1/3; 0.84 to 90.6%); and sterile fluid, 0% (0/2; 0 to 84.2%). For urine samples, AMD had a positive predictive value of 94% (32/34; 79.7 to 98.5%) for diagnosing melioidosis in our cohort. AMD sensitivity on stored sera, collected prospectively from melioidosis cases during this study, was 13.9% (5/36; 4.7% to 29.5%) compared to blood culture samples taken on the same day. In conclusion, AMD is an excellent tool for rapid diagnosis of melioidosis from turbid blood cultures and maintains specificity across all sample types. It is a promising tool for urinary antigen detection, which could revolutionize diagnosis of melioidosis in resource-limited settings. Further work is required to improve sensitivity on nonblood culture samples.

## INTRODUCTION

Burkholderia pseudomallei is a saprophytic bacterium causing melioidosis, a disease with diverse clinical manifestations, including fulminant septicemia, pneumonia, meningo-encephalitis, abscess formation, septic arthritis, and more-indolent cutaneous presentations (1). The global burden of melioidosis has been estimated at 165,000 cases/year with 89,000 fatalities (2). The highest known burden of disease is in south and southeast Asia, where case fatality rates reach around 40% (3), and even in countries with advanced health care systems such as Australia, mortality reaches 14% (4). In the Lao People's Democratic Republic (Laos), increasing numbers of cases have been recognized since the disease was first diagnosed in 1999 (5), and more than 100 cases of culture-confirmed melioidosis are now being identified at Mahosot Hospital Microbiology Laboratory each year with peak incidence during the rainy season (6).

Culture is currently the gold standard diagnostic test for melioidosis, with 100% specificity but an estimated sensitivity of only 60% (7). However, culture confirmation takes a minimum of 48 to 72 h (7) and requires specific media for optimal sensitivity in nonsterile samples (8) and laboratory containment and expertise often not available in areas of endemicity. B. pseudomallei is intrinsically resistant to many antibiotics, and therefore melioidosis does not respond to most agents used for empirical treatment of sepsis, pneumonia, and abscesses in developing countries (9). Therefore, life-saving treatment is often fatally delayed if a specific diagnosis cannot be confirmed. Simple, rapid diagnostic tests for melioidosis for use directly on clinical samples are needed, not only for improving patient outcomes but also to improve epidemiological surveillance of melioidosis and thereby strengthen public health interventions. Immunofluorescence to detect B. pseudomallei directly in clinical samples (10) and latex agglutination for rapid identification of positive cultures (11, 12) are used in some areas where melioidosis is endemic. However, these tests require equipment, expertise, and reagents that are not widely available. More recently, an immunochromatographic lateral flow rapid diagnostic test (RDT) to detect B. pseudomallei extracellular polysaccharide antigen directly in clinical samples has been developed (Active Melioidosis Detect [AMD]; InBios, USA) (13). The method is simple, rapid (results are available within 15 min), and relatively inexpensive (estimated cost, $2/test) and does not require additional equipment; in addition, the kit may be stored at room temperature, making it ideal for use in resource-limited settings. To date, this kit has undergone only limited clinical evaluations (13, 14). We evaluated the diagnostic performance of AMD on a variety of clinical samples (including blood culture broths, pus, sterile fluid, sputum, and urine) over two rainy seasons from patients with suspected and culture-confirmed melioidosis presenting to Mahosot Hospital, Vientiane, Laos.

## MATERIALS AND METHODS

### Analytical sensitivity and specificity.

The limit of detection of the AMD test was assessed using seeded blood cultures and quantitative culture techniques. A single colony of B. pseudomallei (clinical isolate) was taken from a 24- to 48-h culture, and a 0.5 McFarland standard suspension was made using 5 ml phosphate-buffered saline (PBS). This was diluted to 1:1,000,000, and then 1 ml was inoculated into each of 4 negative blood culture bottles (Pharmaceutical factory No. 2, Vientiane, Laos [15]), and the bottles were shaken gently to mix and incubated aerobically at 35 to 37°C. From the time of inoculation (time zero) and every 12 hours thereafter, blood culture broths were observed for turbidity, Gram staining was performed using standard methods, and AMD and quantitative culture were performed. AMD was performed in duplicate on paired uncentrifuged and centrifuged samples from each blood culture broth as follows: 0.5 ml of blood culture broth mixture was dispensed into a 1.5-ml Eppendorf tube, 1 drop of lysis buffer was added, and the suspension was gently mixed using a micropipette. A 20-μl volume of this suspension was then applied to the AMD test strip, followed by 3 drops of chase buffer. The result was read after 15 min according to the manufacturers' instructions (Appendix 1). For centrifuged samples, 1 ml of blood culture broth was centrifuged at 845 × *g* (3,000 rpm on microcentrifuge 5424 from Eppendorf) for 10 min, after which the pellet was resuspended in 1 drop of lysis buffer, and 20 μl of this suspension was applied to the AMD test strip and read as described above. All centrifugation was carried out under biosafety containment level 3 conditions.

Quantitative culture was performed using the Miles et al. method (16): 1 ml was removed from each of the seeded blood culture broths after 0, 12, and 24 h of incubation. Each 1 ml was diluted 1:10 in PBS 8 times to make dilutions of 10^−1^ to 10^−8^ concentrations. A 20-μl volume of each of these dilutions was pipetted onto 1/6 of a blood agar plate, allowed to dry for 20 to 30 min, and then incubated aerobically at 35 to 37°C for 24 h. Each dilution was tested in triplicate. The CFU per milliliter was calculated for each seeded blood culture as the average number of colonies for the dilution where the highest numbers of discrete colonies (between 10 and 100) were clearly seen, multiplied by 50× dilution factor. The result of the quantitative culture was recorded and compared to AMD positivity across the panel of 4 seeded blood cultures to estimate the limit of detection of AMD.

To investigate analytical specificity, negative blood culture broths were seeded (as above) with a range of NCTC/ATCC reference organisms (Escherichia coli ATCC 25922, Klebsiella pneumoniae ATCC 700603, Klebsiella oxytoca NCTC 8167, Enterobacter aerogenes NCTC 10006, Enterobacter cloacae NCTC 11580, Citrobacter freundii NCTC 9750, Edwardsiella tarda NCTC 10396, Salmonella enterica serovar Typhi NCTC 786, S. enterica serovar Enteritidis ATCC 13076, Pseudomonas aeruginosa ATCC 27853, Acinetobacter baumannii NCTC 12156, Ochrobactrum anthropi NCTC 12168, Aeromonas hydrophila NCTC 8049, Yersinia enterocolitica NCTC 11175, Vibrio cholerae NCTC 8021, Burkholderia thailandensis NR-9908, Burkholderia cepacia NCTC 10743, Staphylococcus aureus ATCC 29213, Staphylococcus epidermidis NCTC 11047), soil isolates of Burkholderia thailandensis, and Lao clinical isolates of B. pseudomallei and Burkholderia cepacia (see Table S2 in the supplemental material). Broths were incubated aerobically at 35 to 37°C and observed daily for turbidity. AMD was performed on uncentrifuged samples from turbid blood culture broths (as previously described).

### Prospective evaluation. (i) Study population.

Between 26 June and 18 December 2014, all turbid blood cultures and pus and sputum samples received at Mahosot Hospital Microbiology Laboratory, Vientiane, Laos, were included. Sterile fluid samples received between 10 October and 18 December 2014 were also included. All routine urine samples received between 2 July and 2 September 2014 were included in order to provide baseline specificity data. After an interim analysis, selected urine samples only were included (3 September to 18 December 2014 and 23 June to 12 November 2015). The criterion for selection was the presence of ≥1 of the following clinical details: suspected or confirmed melioidosis; diabetes mellitus and sepsis or fever; prostatitis; or lung, liver, or spleen abscess. Only the first urine sample received from a patient was included.

Stored admission sera from patients diagnosed with melioidosis by culture from any sample between 26 June and 18 December 2014 were retrospectively tested by AMD in June 2015.

### Sample processing.

All samples were processed for microscopy and culture according to standard laboratory procedures (CLSI). B. pseudomallei was isolated from clinical samples using standard media (goat blood, chocolate, and MacConkey agars) and specific selective media (Ashdown's agar and modified Ashdown's selective broth [8]). Each set of blood cultures sent from a patient at any one time contained two blood broth culture bottles (Pharmaceutical factory No. 2, Vientiane, Laos [15]), which were incubated aerobically at 35 to 37°C and observed daily for turbidity for up to 7 days.

### Blood cultures.

AMD was performed on all turbid blood culture broths. Turbid blood cultures with Gram-negative bacilli seen on microscopy also underwent B. pseudomallei latex agglutination and immunofluorescence assay (IFA) testing. The AMD method was as follows: 0.5 ml of turbid blood culture broth was dispensed into a 1.5-ml Eppendorf tube, 1 drop of lysis buffer was added, and the suspension was gently mixed using a micropipette; 20 μl of suspension was added to 3 drops of chase buffer in a 0.6-ml Eppendorf tube and mixed by gentle pipetting. The AMD test strip was then inserted, and the result was read after 15 min according to the manufacturer's instructions (Appendix 1). Latex agglutination was performed as previously described (17): 5 μl of latex reagent was mixed with 1 drop of uncentrifuged blood culture broth on a glass slide, which was gently rocked and observed for agglutination within 2 min. Positive (heat-killed B. pseudomallei) and negative (heat-killed B. thailandensis) controls were performed each day by mixing 5 μl control with 5 μl of latex reagent.

IFA, based on a previously described method (18), was performed as follows: 10 μl of turbid blood culture broth was spread on a glass slide to create a thin smear and allowed to air dry. The slide was then fixed by flooding with absolute methanol for 10 min at room temperature and again allowed to air dry. Ten microliters of IFA reagent (containing 5 μg/ml monoclonal antibody 4B11 and 20 μg/ml Alexa Fluor 488-conjugated goat anti-mouse IgG) was then applied to the smear, and the slide was covered with a coverslip and incubated at room temperature for 5 min before observation under a fluorescence microscope (Nikon Eclipse E600 microscope with the U-FL epifluorescence attachment) at a magnification of ×1,000 using oil immersion. A periphery of bacilli showing strong apple-green fluorescence was recorded as a positive result (10). Positive results were semiquantified using a scheme adapted from the International Union against Tuberculosis and Lung Disease (IUATLD) guidelines for quantification of acid-fast bacilli (AFB) (19) (see Table S3 in the supplemental material). A minimum of 100 fields were examined before slides were recorded as IFA negative if no apple-green fluorescent bacilli were seen. IFA was performed on the same day that the smear was made whenever possible or the next day if not. Slides of heat-killed (1 × 10^6^ CFU/ml in PBS at 80°C for 1 h) B. pseudomallei and B. thailandensis were used as positive and negative controls, respectively, for each batch of immunofluorescence tests. Routine laboratory staff performed the latex agglutination and AMD tests, while all IFA tests were performed by L. Boutthasavong, blinded to the latex and AMD test results. All blood culture work was performed in a biosafety cabinet at laboratory containment level 2 according to normal local practice.

### Pus, sputum, and sterile fluid.

AMD and IFA were performed on all pus, sputum, and sterile fluid samples on receipt in the laboratory. Latex agglutination is not validated for these sample types and was therefore not performed on these samples. IFA was performed as described above using 1 μl of sputum, pus, or sterile fluid sample to make the original smear. AMD was performed as follows: 20 μl of pus, sterile fluid, or thick/viscous sputum samples was mixed with 3 or 4 drops of lysis buffer by vortexing for 15 s. Twenty microliters of this mixture was then added to 3 drops of chase buffer in a 0.6-ml Eppendorf tube and mixed by gentle pipetting. An AMD test strip was inserted, and the result was read after 15 min. Thin/watery sputum samples were processed in the same way, except that 50 μl of sample was mixed with 2 or 3 drops of lysis buffer initially. Routine laboratory staff performed AMD tests, while IFA was performed by L. Boutthasavong, blinded to the results of AMD.

### Urine.

AMD was performed using 50 μl of neat urine, which was added to 3 drops of chase buffer in a 0.6-ml Eppendorf tube and mixed by gentle pipetting. An AMD test strip was then inserted, and the result was read after 15 min. AMD tests were performed by routine laboratory staff. If the AMD result for neat urine was negative, then urine concentration was performed when concentrators (Minicon B15; Merck Millipore Ltd.) were available. Five milliliters of urine was concentrated 100 times by using these simple tabletop concentrators according to the manufacturer's instructions. AMD was then performed as described above using 20 μl of concentrated urine. Neither latex agglutination nor IFA has been validated for use directly with urine samples, and therefore neither of these tests was performed on these samples.

### Sera.

Admission sera (stored at −80°C) from culture-confirmed melioidosis cases were retrieved, and AMD was performed as follows: 35 μl serum was added to the AMD test strip, followed by 3 drops of chase buffer. Results were read after 15 min as described previously.

Each new box of AMD test strips underwent quality assurance by testing one strip from the box as follows: a single colony of a B. pseudomallei-positive control (Lao clinical isolate UI 8976) was emulsified, using a sterile loop, in 2 drops of lysis buffer. Three drops of chase buffer was then added to this bacterial suspension and mixed gently by pipetting before the AMD test strip was inserted and read as previously described.

### Data analysis.

The diagnostic sensitivity and specificity of the AMD, latex agglutination, and IFA on the different sample types were calculated using culture as the reference standard. The sensitivity and specificity of AMD were compared with those of latex agglutination and IFA on turbid blood cultures containing Gram-negative bacilli and with those of IFA alone for pus, sputum, and sterile fluid samples using a two-sample test of proportions. In patients confirmed with melioidosis (by culture from any sample) whose urine was culture negative for B. pseudomallei, a two-sided Fisher's exact test was used to analyze the association between urine AMD positivity and (i) disseminated versus localized melioidosis and (ii) presence or absence of B. pseudomallei bacteremia. Melioidosis cases were defined as “disseminated” when B. pseudomallei bacteremia was present and/or there was clinical/radiological or microbiological evidence of multiple sites of disease. Cases were defined as “localized” if only one site of disease was present, e.g., pneumonia or parotitis. Analysis was done using STATA, v14.2 (College Station, TX, USA).

## RESULTS

### Analytical sensitivity and specificity.

All B. pseudomallei-seeded blood cultures were AMD positive by 12 h of incubation (see Table S1 in the supplemental material). The lower limit of detection of the AMD was found to be 1.4 × 10^5^ CFU/ml, meaning that it was positive in 3 of 4 seeded blood cultures before they became visibly turbid and in 4 of 4 seeded blood cultures before organisms were detectable by Gram staining of the broth. Centrifugation of blood culture broth prior to performing AMD did not reduce time to positivity.

All non-B. pseudomallei reference strains were negative by AMD (see Table S2 in the supplemental material). Two soil isolates of B. thailandensis and one sputum isolate of B. cepacia were AMD positive. All 3 of these isolates were known to give a positive B. pseudomallei latex agglutination test, probably due to production of a cross-reacting extracellular polysaccharide (20). All four positive-control B. pseudomallei clinical isolates were positive by AMD.

### Prospective evaluation.

Between 26 June and 18 December 2014, 89 patients were diagnosed with melioidosis by culture of B. pseudomallei from at least one sample type (blood, pus, sputum, sterile fluid, urine, throat swab, wound swab [see Fig. S1 in the supplemental material]). The median age was 45 years (interquartile range [IQR], 27 to 54 years), 55% were male, and 20/89 (22.5%) patients died while in the hospital or were discharged moribund. Blood cultures were received from 85 of these patients (85/89, 96%) of whom 54% (46/85) were bacteremic with B. pseudomallei. Of the 43 patients not found to be bacteremic, 34 had localized disease while 9 had evidence of multifocal disease.

### Turbid blood cultures.

**FIG 1:**
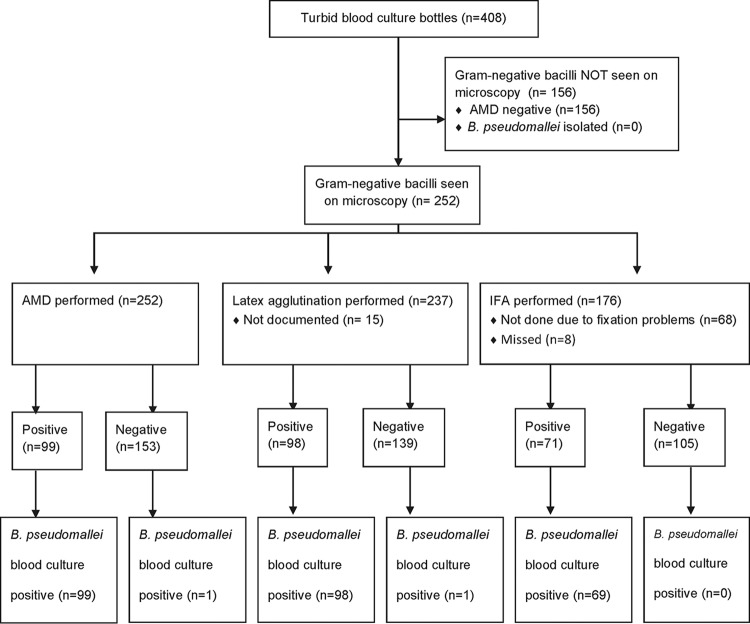
Sample flow diagram of turbid blood cultures with Gram-negative bacilli seen on microscopy. In this and all subsequent figures, “not documented” means that there was inadequate documentation to include the result in the analysis; “missed” means that the test was not performed.

There were 412 turbid blood culture bottles during the study period, of which 4 did not have AMD performed and were therefore excluded; 408 turbid blood culture bottles from 247 patients were thus included in the analysis. Organisms isolated from the 408 blood cultures are shown in Fig. S2 in the supplemental material. B. pseudomallei was isolated from 100 bottles (Fig. 1). The overall AMD sensitivity was 99% (99/100; 95% confidence interval, 94.6 to 100%) and specificity was 100% (308/308; 98.8 to 100%). Of these 408 turbid blood culture bottles, 252 had Gram-negative bacilli (GNB) seen on Gram stain and therefore had additional rapid tests performed: latex agglutination (*n* = 237) and immunofluorescence assay (IFA; *n* = 176) (Fig. 1). Sensitivities and specificities for these tests are given in Table 1. A total of 166 turbid blood culture samples with GNB seen on microscopy had all 3 rapid tests performed, with agreement between all 3 rapid tests and culture for 98.2% of them (163/166). One sample was AMD and latex agglutination negative but IFA and B. pseudomallei culture positive. Two samples were scanty positive by IFA (1 to 3 bacilli/100 fields) but negative by all other tests.

**TABLE 1:** Diagnostic sensitivity and specificity for all tests and sample types compared with culture as the reference standard^*a*^

Specimen type (*n*)	Test (*n*)	% Sensitivity (95% CI)	% Specificity (95% CI)
Turbid blood culture with GNB (252)	AMD (252)	99.0 (94.6–100)	100 (97.6–100)
	Latex (237)	99.0 (94.5–100)	100 (97.4–100)
	IFA (176)	100 (94.8–100)	98.1 (93.4–99.8)
	*P* value	NS*	NS**
Pus (139)	AMD (139)	47.1 **(**23.0–72.2)	100 (97.0–100)
	IFA (95)	66.7 (29.9–92.5)	90.7 (82.5–95.9)
	*P* value	0.338	0.0006
Sputum (23)	AMD (23)	33.3 (0.84–90.6)	100 (83.2–100)
	IFA (23)	100 (29.2–100)	85.0 (62.1–96.8)
	*P* value	0.083	0.072
Sterile fluid (46)	AMD (46)	0 (0–84.2)	100 (92.0–100)
	IFA (43)	100 (15.8–100)	100 (91.4–100)
	*P* value	0.046	1.0
Urine selected (241)	AMD (241)	86.7 (59.5–98.3)	90.7 (86.2–94.2)

a NS, not significant; CI, confidence interval; *n*, number of samples. *, *P* = 0.994 for AMD versus latex, 0.402 for latex versus IFA, 0.405 for IFA versus AMD; **, *P* = 1.0 for AMD versus latex, 0.107 for latex versus IFA, 0.169 for IFA versus AMD.

### Pus.

**FIG 2:**
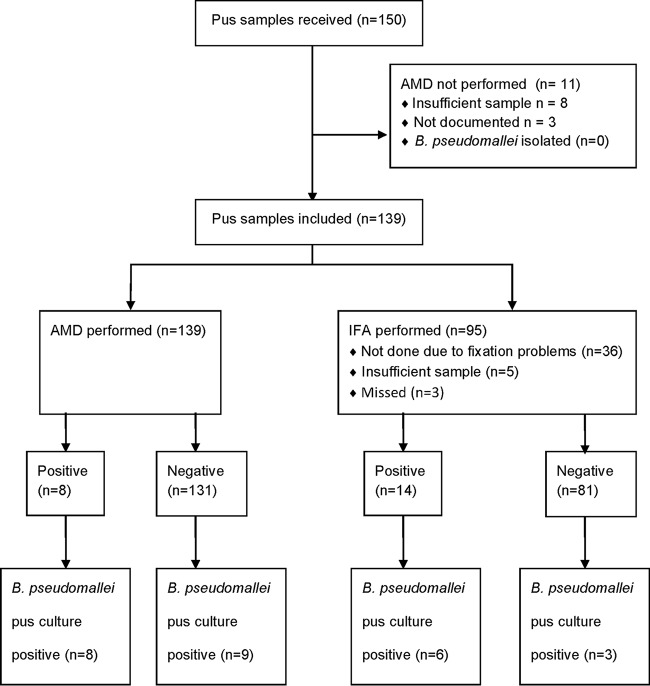
Sample flow diagram for pus samples.

Of 150 pus samples received during the study period, 139 had AMD performed and were included in the analysis. B. pseudomallei was cultured from 17/139 samples. Ninety-five of 139 pus samples also underwent IFA (Fig. 2). AMD was significantly more specific than IFA (*P* = 0.0006; Table 1). However, all 8 false-positive IFA results were reported as scanty (1 to 5 bacilli/100 fields).

### Sputum.

**FIG 3:**
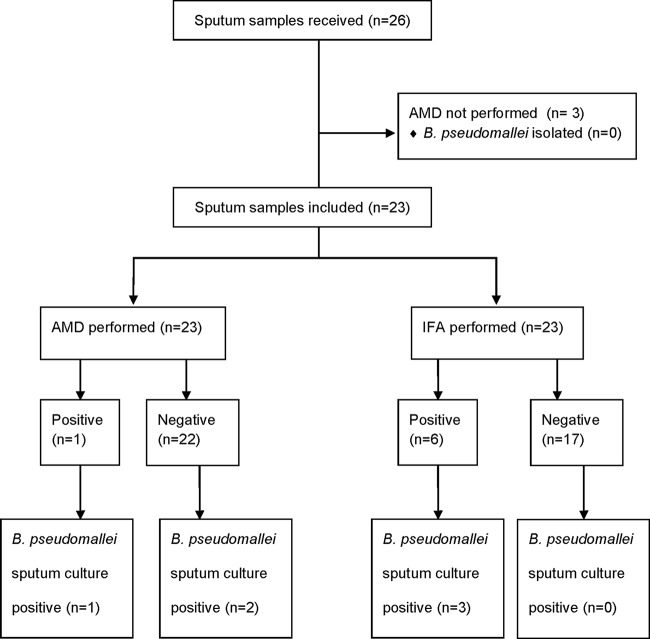
Sample flow diagram for sputum samples.

Twenty-six sputa were received during the study period, 3 of which did not have AMD performed and were excluded. The remaining 23 sputa underwent both AMD and IFA testing, and no significant difference in sensitivity or specificity was found (Table 1). Three of 23 samples were B. pseudomallei culture positive, all 3 of which were IFA positive but only 1 of which was AMD positive (Fig. 3). There were 3 positive IFA tests from culture-negative samples, but all were reported as scanty (1 to 7 bacilli/100 fields), and 2 of these samples were from the same patient, who, although culture negative for B. pseudomallei, had a clinical picture compatible with melioidosis and died shortly after transfer to Thailand for further health care.

### Sterile fluid.

**FIG 4:**
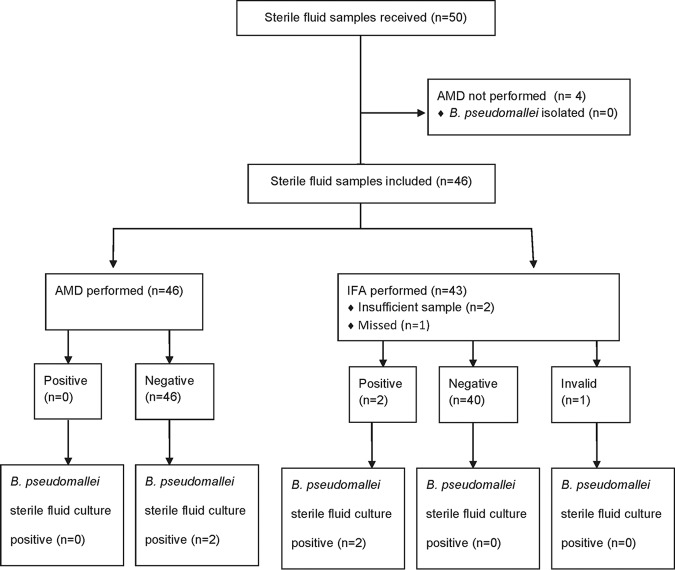
Sample flow diagram for sterile fluid samples.

Between 10 October and 18 December 2014, 50 sterile fluid samples were received, and 46 underwent AMD and were included in the analysis (29 pleural fluid, 6 joint fluid, 2 pericardial fluid, and 9 ascitic fluid smaples); 42 of the 46 also underwent IFA. B. pseudomallei was isolated from 2 samples (both were joint fluid samples from the same patient, taken 11 days apart); IFA was positive on both of these samples, while AMD was negative on both (Fig. 4). The sensitivity of IFA was therefore significantly better than AMD (Table 1), although numbers were small and confidence intervals wide.

### Urine.

**FIG 5:**
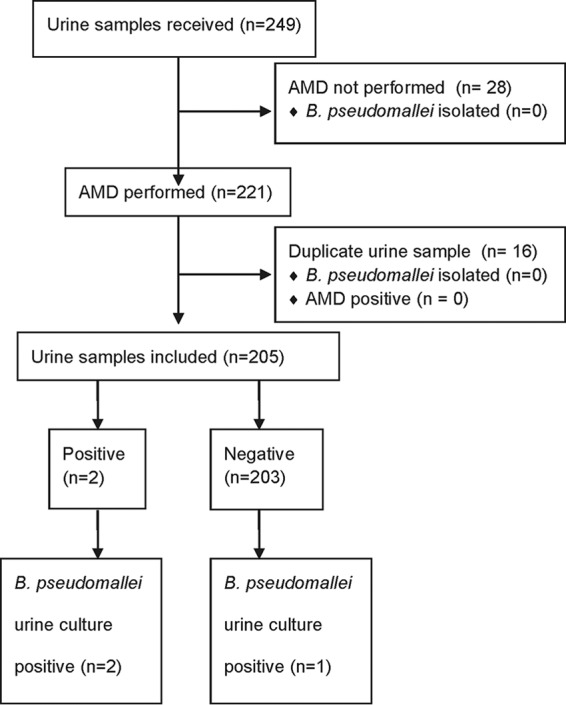
Sample flow diagram for unselected urine samples (2 July to 2 September 2014).

Between 2 July and 2 September 2014, 249 urine samples were received. AMD was not performed on 28 of them, and 16 were duplicate specimens; thus, 205 were included in the analysis (Fig. 5). Three of 205 urine samples were B. pseudomallei culture positive, 2 of which were AMD positive (sensitivity, 66.7% [9.4 to 99.2%]). The B. pseudomallei culture-positive urine sample that was AMD negative had a low bacterial load, with only 1 CFU isolated from a centrifuged deposit. AMD specificity was 100% (98.2 to 100%; 202/202). Organisms isolated in culture from all 205 urine samples are shown in Fig. S3 in the supplemental material.

**FIG 6:**
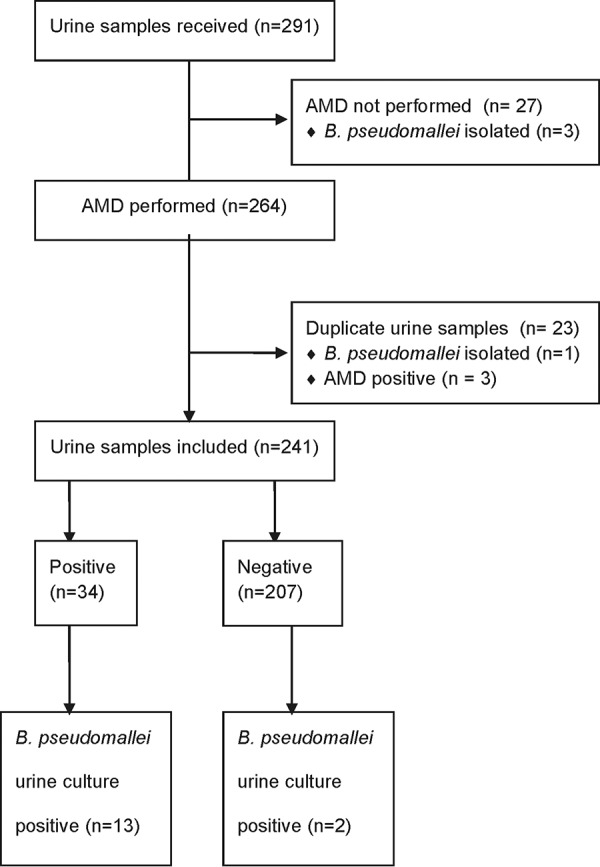
Sample flow diagram for selected urine samples (3 September to 18 December 2014 and 23 June to 12 November 2015).

From 3 September onwards, only selected urine samples were included in the study (3 September to 18 December 2014, *n* = 102; 23 June to 12 November 2015, *n* = 189). AMD was not performed on 27 samples (2014, 5 samples; 2015, 22 samples), and 23 samples were duplicates and therefore excluded. Thus, 241 samples were included in the analysis. Fifteen of 241 urine samples were B. pseudomallei culture positive, 13/15 of which were AMD positive (Table 1). Twenty-one of 226 urine culture-negative samples were AMD positive (Fig. 6). Interestingly, 19/21 of these urine samples came from patients who had melioidosis confirmed by culture from another site, suggesting that these were not “false-positive” AMD results but that the AMD was detecting true B. pseudomallei antigenuria. The positive predictive value of AMD on urine for correctly diagnosing melioidosis in this cohort was therefore 94.1% (32/34 [79.7 to 98.5%]) with a disease prevalence of 35.7% (86/241).

### Urine samples from melioidosis cases.

To further describe B. pseudomallei antigenuria in our cohort, results of urine samples received from the 182 culture-confirmed (from any site) melioidosis cases during the 2014 and 2015 study periods were analyzed further. One urine sample (each) was received from 114 (2014, 57; 2015, 57) of these 182 patients, and 20/114 urine samples were culture positive for B. pseudomallei (16/20 were AMD positive). Twenty-one of the 94 culture-negative urine samples were AMD positive (Table 2). Patients who were urine culture negative for B. pseudomallei but had disseminated melioidosis were significantly more likely to be urine AMD positive than those with localized melioidosis (18/61 versus 3/32; *P* = 0.036). The presence of B. pseudomallei bacteremia did not increase the likelihood of urine AMD positivity overall (15/49 versus 6/44, *P* = 0.08) or in patients with disseminated melioidosis (15/49 versus 3/12, *P* = 1.0).

**TABLE 2:** Urine AMD results according to site of disease in melioidosis cases that were urine culture negative for *B. pseudomallei*^*a*^

Site of disease (*n*)	No. of cases with urine AMD result:	
	Positive	Negative
Disseminated (61)		
Bacteremic (49)	15	34
Not bacteremic (12)	3	9
Localized (32)	3	29

a *n*, number of patients. The total number of patients was 93; 1 patient did not have sufficient data available to categorize the site of disease.

### Urine concentration.

Due to the lack of availability of urine concentrators, the urine concentration assay was performed retrospectively on 20 stored urine isolates collected prospectively between 2 July and 18 December 2014. All urine samples were from confirmed melioidosis cases but were AMD negative on neat urine and B. pseudomallei urine culture negative. Six of 20 urine samples were AMD positive after urine concentration.

### Sera.

**FIG 7:**
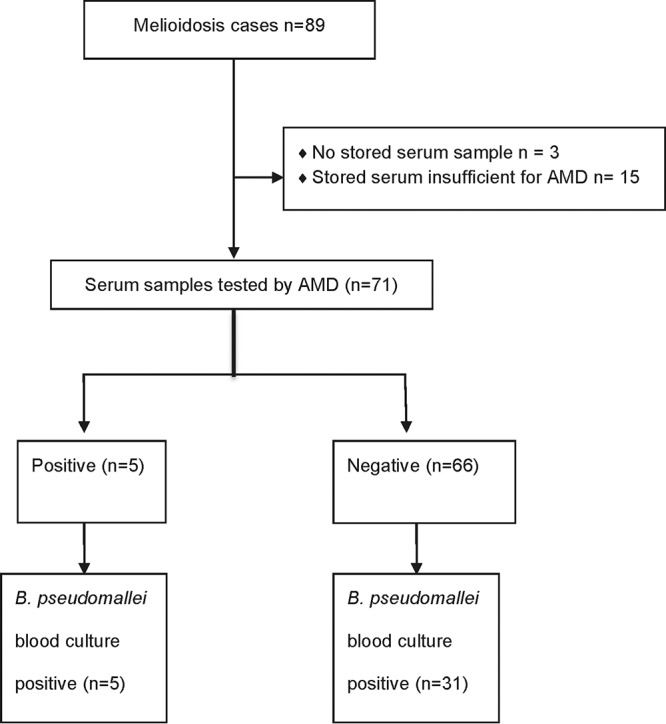
Sample flow diagram for serum samples from culture-confirmed melioidosis cases diagnosed between 26 June and 18 December 2014.

Seventy-one stored serum samples from the 89 melioidosis cases diagnosed from 26 June to 18 December 2014 were available for AMD testing (Fig. 7). Five of 71 (7%) samples were AMD positive. Each of these five patients was culture positive for B. pseudomallei from blood taken on the same day as the serum sample. Of the patients negative by AMD on sera, 31/66 had been bacteremic with B. pseudomallei on the same day as the serum sample was taken, and a further 9 nonbacteremic patients had evidence of disseminated disease. Sera therefore had low sensitivity for diagnosis of melioidosis compared with blood culture as the gold standard, 13.9% (5/36; 4.7% to 29.5%).

## DISCUSSION

We evaluated the accuracy of the AMD lateral flow immunoassay (LFI) for the rapid diagnosis of melioidosis directly from clinical samples. The LFI detects an extracellular polysaccharide of B. pseudomallei with a limit of detection (LOD) of 0.2 ng/ml (13, 21). On turbid blood cultures, the AMD was found to have excellent analytical and diagnostic sensitivity and specificity, comparable to those of both IFA and latex agglutination, which were found to have performance characteristics similar to those published in previous studies (11, 17, 18, 22). One turbid blood culture bottle was AMD and latex agglutination negative but culture positive for B. pseudomallei. After 24 h of further incubation, both tests were positive on the same bottle, suggesting that the initial false-negative results may reflect an initial bacterial load lower than the limit of detection for both tests. The high specificity of the AMD test on turbid blood cultures is extremely promising for deployment to field settings in the tropics, where simple tests such as AMD may be performed after a broth incubation step in the absence of laboratory facilities for Gram stain and culture. A positive AMD result, potentially obtained as early as 12 h following blood sampling, could prove to be life-saving if antimicrobial treatment is adapted appropriately.

The AMD test maintained excellent analytical and diagnostic specificity across all sample types, even “nonsterile” samples such as sputum and urine, which are more likely to contain contaminating bacteria (including environmental Burkholderia spp. in a tropical setting of endemicity). False-positive AMD results were seen in our study only in the case of environmental Burkholderia strains known to express an extracellular polysaccharide similar to that detected by the AMD. Although this may pose a problem when AMD is used for detection of B. pseudomallei in environmental samples such as soil (23), in clinical samples it is rarely likely to be relevant. The finding of AMD reactivity in a clinical strain of Burkholderia cepacia in this study is novel, as previous testing of B. cepacia complex strains had not demonstrated this (D. P. AuCoin, unpublished observations). Relatively few sputum samples were included in this study, and further work is needed to investigate how frequently respiratory samples containing non-pseudomallei Burkholderia (for example, from patients with cystic fibrosis) may cause AMD reactivity. However, in our experience, such strains are rare and the majority of B. cepacia isolates do not cross-react in this way (data not shown).

AMD analytical sensitivity was extremely promising, with AMD LOD on broth cultures better than previous estimates of LOD for latex agglutination (1 × 10^6^ to 2 × 10^6^ CFU/ml [22]) and similar to estimated bacterial loads of B. pseudomallei in urine (1.5 × 10^4^ CFU/ml), sputum (1.1 × 10^5^ CFU/ml), and pus (1.1 × 10^7^ CFU/ml) samples (24). However, diagnostic sensitivity was disappointing in nonblood culture samples. We observed that viscous samples were more challenging to process for AMD, and this, coupled with low initial bacterial loads, may explain the moderate sensitivity observed for the test in these samples. Seven pus samples in our study were received in broth from distant study sites (and therefore excluded from the main analysis), and AMD was positive in all 3 samples that were subsequently B. pseudomallei culture positive. An initial enrichment culture step has been previously suggested to improve the sensitivity of IFA for B. pseudomallei from nonblood clinical samples (10). It is likely that broth incubation of pus, sputum, and sterile fluid found to be AMD negative on direct testing will increase AMD sensitivity on these samples, while only delaying diagnosis by a few hours, although appropriate laboratory biosafety equipment and practices would be needed.

IFA diagnostic sensitivity and specificity in this study were similar to those published in previous reports (10). However, despite the reportedly lower LOD (2 × 10^3^ CFU/ml of IFA [10]), IFA was not found to be significantly more sensitive than AMD on any sample type except sterile fluids, nor was IFA significantly more specific than AMD except on pus samples. The inherent subjectivity of immunofluorescence microscopy, the possibility of misidentifying fluorescent debris as bacteria, and the labor- and resource-intensive methodology are important disadvantages of IFA compared with AMD.

The number of B. pseudomallei culture-positive urine samples in this study was limited; however, the ability of AMD to detect B. pseudomallei antigenuria in melioidosis patients, particularly those with disseminated melioidosis whose urine is culture negative for B. pseudomallei, is encouraging. Our findings replicate previous work in a nonhuman primate model, in which antigen was detectable in urine by AMD as early as 2 to 3 days after experimental infection with B. pseudomallei (D. P. AuCoin, unpublished). The extracellular polysaccharide detected by AMD maintains its molecular weight in urine without degradation over time, which might otherwise affect the sensitivity of AMD on urine (25). Urine is an easily available, noninvasive sample and a simple matrix for AMD. Reliable urine antigen detection for melioidosis by a rapid test such as AMD could revolutionize diagnostics in resource-limited parts of the world, where this disease is most prevalent. However, the overall sensitivity of urine AMD for detection of melioidosis in our cohort was only 33% (37/114; 24 to 41.9%). The use of simple tabletop urine concentrators increased AMD sensitivity in this study; however, the numbers tested were limited and further work is needed to establish the extent to which urine concentration improves AMD sensitivity in urine. At approximately $6/sample, cost may prove a barrier to the use of this concentration technique for urine samples in settings of endemicity.

The AMD test is an extremely promising tool for diagnosis of melioidosis worldwide and meets all affordable, sensitive, specific, user friendly, rapid and robust, equipment-free, and delivered (ASSURED) criteria for RDTs (26), although further work is required to optimize sensitivity on nonblood samples. However, it is not yet a true “point of care” test, with AMD sensitivity on stored whole-blood samples from bacteremic melioidosis patients having been shown to be 40% (16/40; compared with 20% sensitivity for molecular detection on the same samples) previously (14) and on stored serum samples in this study only 7% (5/71). The low bacterial load in blood samples (1 CFU/ml prior to broth incubation [13]) may limit the utility of the AMD test on these sample types, and further large-scale prospective evaluations are required. However, the simple nature of the AMD technology means that it is more likely than molecular diagnostics to be widely applicable in a developing country context for the foreseeable future.

The main limitation of this study was that not all samples underwent testing by all the relevant rapid tests. This was partly related to the nature of the study, with the assays being performed during routine processing in a busy diagnostic laboratory. However, this study design gave a realistic indication of assay performance in a routine setting. In addition, the immunofluorescence assay, which had not previously been routinely used in our laboratory, took longer than expected to optimize, and therefore turbid blood cultures and pus received in the initial stages of the study did not undergo IFA. Sputum and sterile fluid analyses were also limited due to low sample numbers.

In some patients in our study, a positive urine AMD result was the first indication of melioidosis, preceding culture confirmation by at least 24 h and resulting in an early switch to appropriate antibiotics in critically unwell patients. This study was not designed to evaluate the clinical impact of obtaining a rapid diagnosis of melioidosis by AMD; however, now that we have shown the diagnostic specificity of this test to be excellent, we are further investigating this. Throat swabs are another easily obtained sample type that is routinely used for diagnosis of melioidosis by culture in our setting. Incubation of a throat swab in liquid selective enrichment broth such as SBCT (8) would enable throat swabs to be used for rapid diagnosis of melioidosis by AMD, and this also warrants further study.

In conclusion, the AMD has excellent sensitivity and specificity for early detection of B. pseudomallei in blood culture broth. It also has the advantage over the latex agglutination test that it can be used directly on other sample types for the diagnosis of melioidosis. Specificity is retained when used directly on these other samples, but sensitivity is only moderate and requires optimization. The ability to identify melioidosis from urine samples by using AMD may significantly enhance diagnosis of this neglected disease. Studies are needed in a variety of different prevalence settings in order to truly understand the utility of this assay; however, deployment of the AMD test globally might improve our understanding of the epidemiology of melioidosis. Ultimately, we hope to see LFI technology used for multiplex antigen detection of a number of important causes of febrile illness in resource-limited settings.

## Supplementary Material

Supplemental material

## Figures and Tables

**FIG A1:**
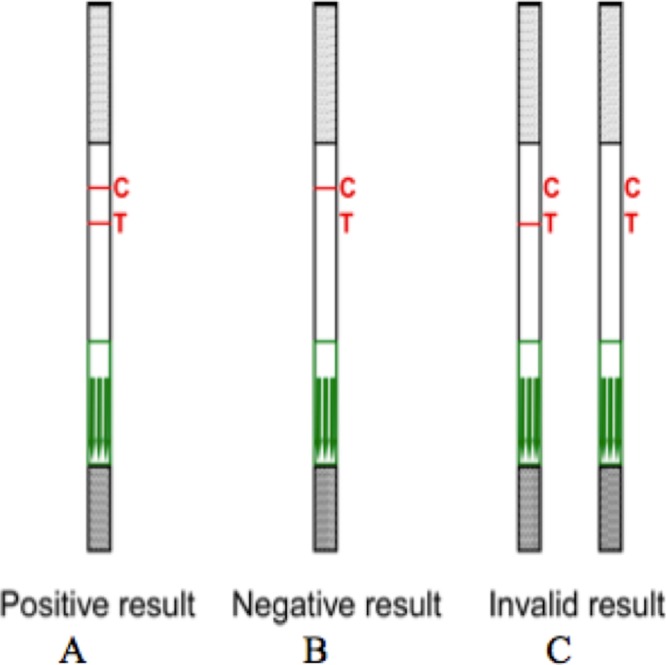
Interpretation according to the instructions of the manufacturer (InBios). C, control line; T, test line.

**FIG A2:**
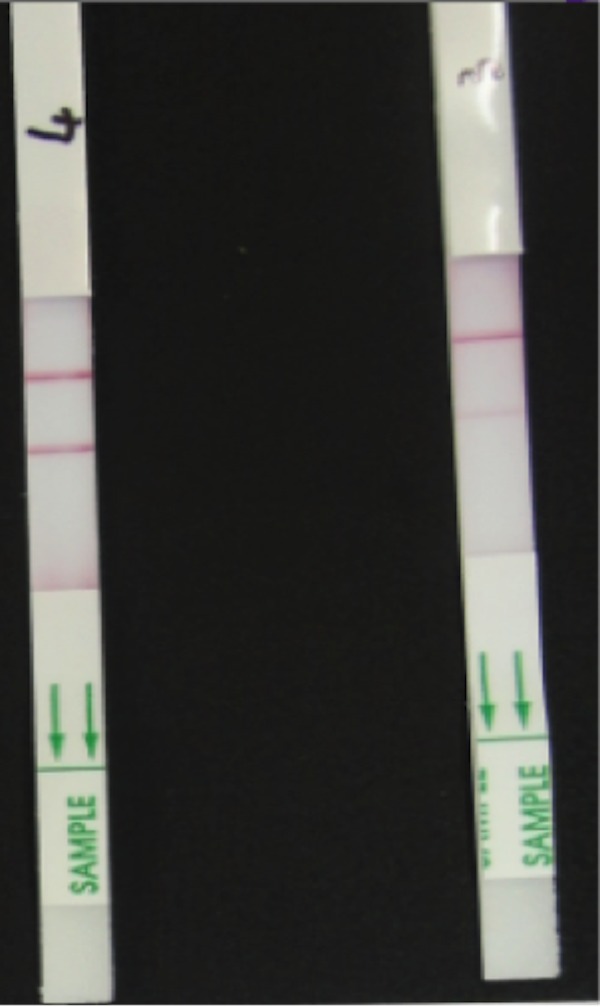
Positive results.

## APPENDIX 1

### Interpretation of AMD reactivity.

In Fig. A1, interpretation according to the manufacturer's instructions is shown. Note that a faint test line is considered positive, as the red color in this region varies depending on the concentration of antigen present. Examples of positive AMD results are given in Fig. A2.
