# Supplementary material for: Evaluation of a Rapid Diagnostic Test for Detection of Burkholderia pseudomallei in the Lao People's Democratic Republic
Source: J Clin Microbiol. 2018 Jun 25;56(7):e02002-17. doi: 10.1128/JCM.02002-17 (PMC6018328; doi:10.1128/JCM.02002-17)
Supplement: Supplemental material [file supp_56_7_e02002-17__index.html]

Evaluation of a Rapid Diagnostic Test for Detection of Burkholderia pseudomallei in the Lao People's Democratic Republic — Supplemental material 

# Evaluation of a Rapid Diagnostic Test for Detection of Burkholderia pseudomallei in the Lao People's Democratic Republic

## Supplemental material

- Supplemental file 1 -

  Tables S1 (Analytical sensitivity), S2 (Analytical specificity), and S3 (Schema for quantification of positive IFA results) and Fig. S1 (Sample types culture positive for *B. pseudomallei* in confirmed melioidosis cases from June to December 2014), S2 (Organisms isolated from turbid blood culture broths from 26 June to 18 December 2014), and S3 (Organisms isolated from culture of unselected urine samples from 2 July to 2 September 2014)

  PDF, 563K
