# Supplementary material for: Evaluation of a Rapid Diagnostic Test for Detection of Burkholderia pseudomallei in the Lao People's Democratic Republic
Source: J Clin Microbiol. 2018 Jun 25;56(7):e02002-17. doi: 10.1128/JCM.02002-17 (PMC6018328; doi:10.1128/JCM.02002-17)
Supplement: Supplemental material [file JCM.02002-17_zjm999096004s1.pdf]

Seeded Blood Culture	Test	Time		
		0 hrs	12 hrs	24 hrs
1	Turbid	No	No	No
	Gram stain	NOS	NOS	GNR
	AMD neat	-	weak +	+++
	AMD centrifuged	-	weak +	+++
	Quantitative culture (CFU/ml)	0	1.4×10^{5}	7.3×10^{7}
2	Turbid	No	Yes	Yes
	Gram stain	NOS	NOS	GNR
	AMD neat	-	weak +	++
	AMD centrifuged	-	+	+++
	Quantitative culture (CFU/ml)	0	2.1×10^{6}	3.1×10^{8}
3	Turbid	No	No	Yes
	Gram stain	NOS	NOS	GNR
	AMD neat	-	weak +	++
	AMD centrifuged	-	+	+++
	Quantitative culture (CFU/ml)	0	1.8×10^{6}	1.6×10^{8}
4	Turbid	No	No	No
	Gram stain	NOS	NOS	GNR
	AMD neat	-	weak +	++
	AMD centrifuged	-	weak +	+++
	Quantitative culture (CFU/ml)	0	1.7×10^{5}	2.0×10^{8}

Note: NOS = no organisms seen; - = negative; + = positive.

Supplemental Table S1. Analytical sensitivity.

Organism	Reference Strain or Clinical isolate	AMD result
Escherichia coli	ATCC 25922	negative
Klebsiella pneumoniae	ATCC 700603	negative
Klebsiella oxytoca	NCTC 8167	negative
Enterobacter aerogenes	NCTC 10006	negative
Enterobacter cloacae	NCTC 11580	negative
Citrobacter freundii	NCTC 9750	negative
Edwardsiella tarda	NCTC 10396	negative
Salmonella Typhi	NCTC 786	negative
Salmonella Enteritidis	ATCC 13076	negative
Pseudomonas aeruginosa	ATCC 27853	negative
Acinetobacter baumannii	NCTC 12156	negative
Ochrobactrum anthropi	NCTC 12168	negative
Aeromonas hydrophila	NCTC 8049	negative
Yersinia enterocolitica	NCTC 11175	negative
Vibrio cholerae	NCTC 8021	negative
Burkholderia thailandensis	NR-9908 (E426)	negative
Burkholderia cepacia	NCTC 10743	negative
Staphylococcus aureus	ATCC 29213	negative
Staphylococcus epidermidis	NCTC 11047	negative
Burkholderia thailandensis	Soil isolate E555*	positive
Burkholderia thailandensis	Water isolate ST10*	positive
Burkholderia pseudomallei	Clinical isolate (blood culture UI27784)	positive
Burkholderia pseudomallei	Clinical isolate (blood culture UI28310)	positive
Burkholderia pseudomallei	Clinical isolate (blood culture FS4001)	positive
Burkholderia pseudomallei	Clinical isolate (blood culture FS4025)	positive
Burkholderia cepacia	Clinical isolate (sputum 39628**)	positive

*Known *B. pseudomallei* latex agglutination positive **Lao clinical isolate giving false positive latex agglutination result, confirmed as *B. cepacia* complex by API20NE and subsequent 16S sequencing.

Supplemental Table S2. Analytical specificity.

Report as:	X1000 magnification
Scanty	1-9 bacilli per slide/ 100 fields
1+	10-99 bacilli per slide/ 100 fields
2+	1-10 bacilli per field (check 50 fields)
3+	>10 bacilli per field (check 20 fields)

Supplemental Table S3. Schema for quantification of positive IFA results.

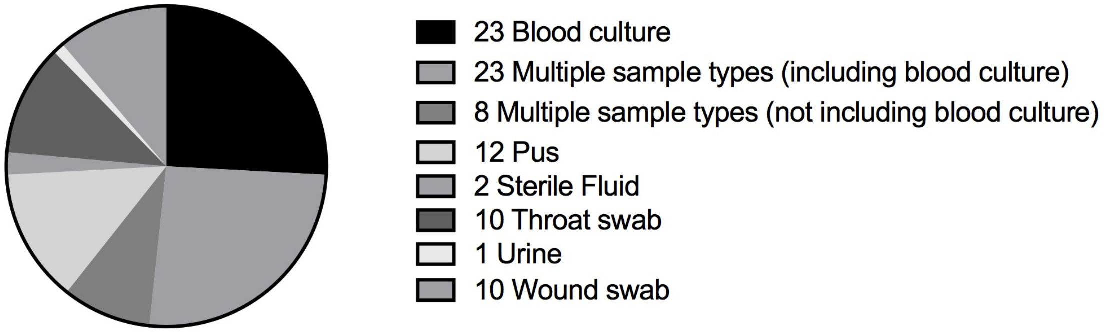

Supplemental Figure S1. Sample types culture positive for *B. pseudomallei* in confirmed melioidosis cases June – December 2014 (N = 89).

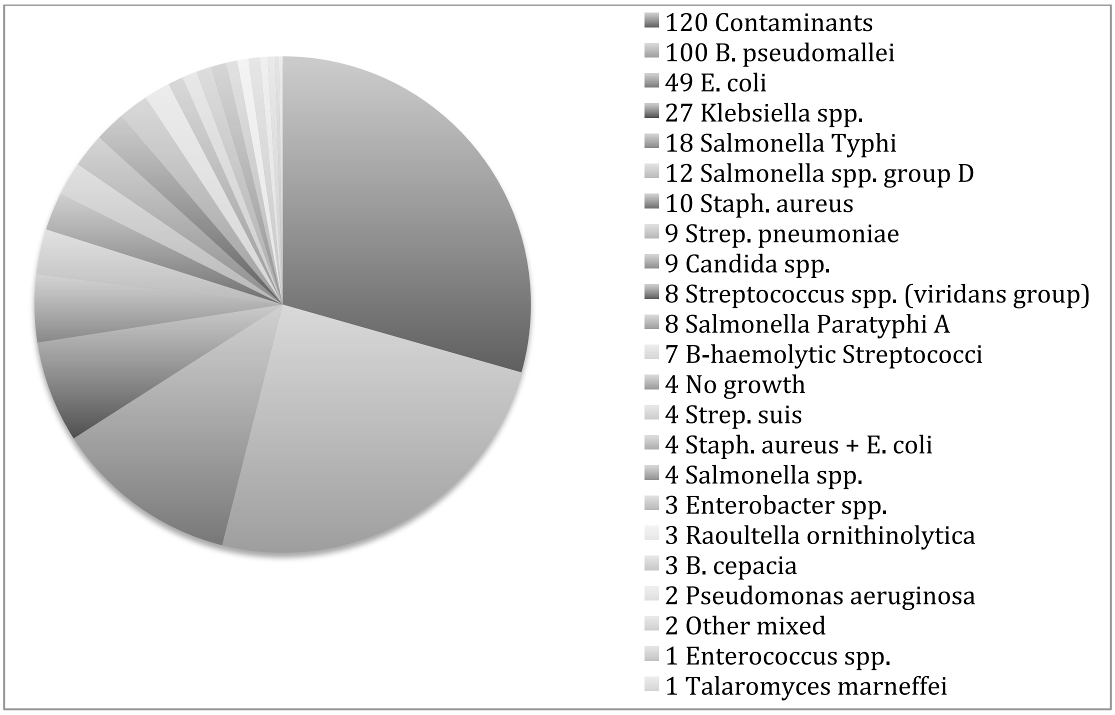

Supplemental Figure S2. Organisms isolated from turbid blood culture broths 26th June – 18th December 2014 (N = 408 blood culture bottles from 247 patients).

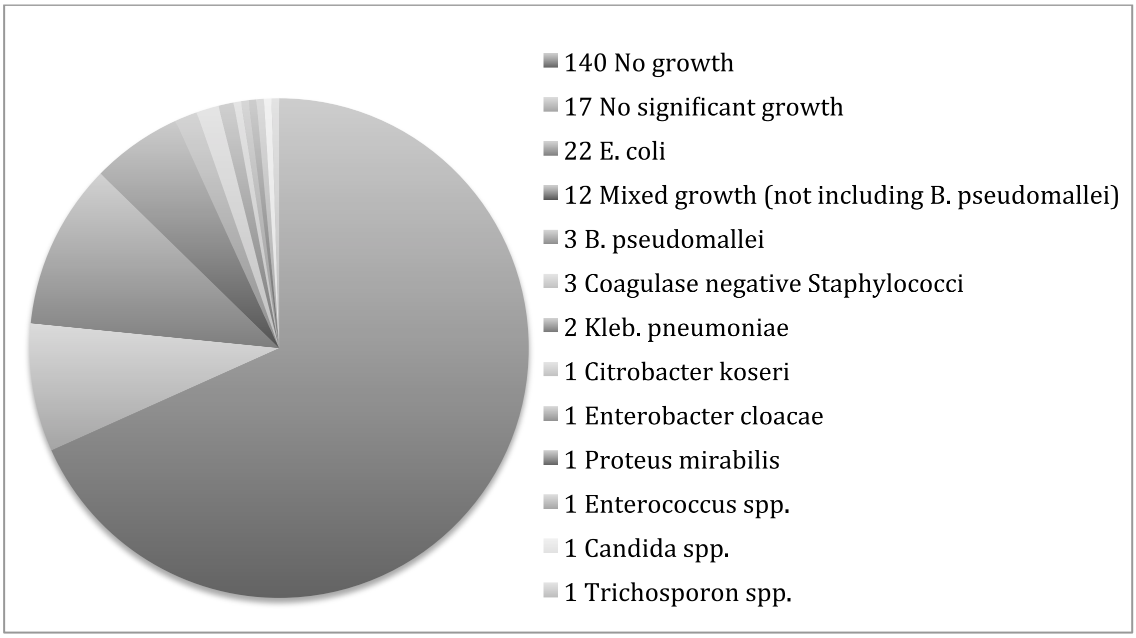

Supplemental Figure S3. Organisms isolated from culture of unselected urine samples 2nd July – 2nd Sept. 2014 (N = 205).
